# Mast cells contribute to alveolar bone loss in Spontaneously Hypertensive Rats with periodontal disease regulating cytokines production

**DOI:** 10.1371/journal.pone.0247372

**Published:** 2021-03-04

**Authors:** Victor Gustavo Balera Brito, Mariana Sousa Patrocinio, Maria Carolina Linjardi Sousa, Ayná Emanuelli Alves Barreto, Sabrina Cruz Tfaile Frasnelli, Vanessa Soares Lara, Carlos Ferreira Santos, Sandra Helena Penha Oliveira

**Affiliations:** 1 Programa Multicêntrico de Pós-graduação em Ciências Fisiológicas, SBFis, São Paulo State University (UNESP), School of Dentistry, Araçatuba, SP, Brazil; 2 Department of Basic Sciences, São Paulo State University (UNESP), School of Dentistry, Araçatuba, SP, Brazil; 3 Department of Biological Science, Bauru School of Dentistry, University of São Paulo (USP), SP, Brazil; 4 Department of Stomatology, Bauru School of Dentistry, University of São Paulo (USP), SP, Brazil; Università degli Studi della Campania, ITALY

## Abstract

Mast cells (MCs) play a pivotal role in inflammatory responses and had been studied in inflammatory bone disorders, however, their role in alveolar bone loss induced by periodontal disease (PD) is not yet fully understood. We, therefore, aimed to evaluate the effects of MCs depletion in the PD-induced alveolar bone loss in Wistar (W) and Spontaneously Hypertensive Rats (SHRs). PD was induced by ligating the lower first molars with silk thread one day after the MCs depletion, by the pre-treatment with compound 48/80 for 4 days. After 15 days of PD induction, the hemi-mandibles were surgically collected for qRT-PCR, histological analyses, immunostaining, and ELISA. Systolic blood pressure (SBP) was verified by tail plethysmography to confirm the hypertensive status, and SHR presented SBP >150 mmHg, and previous MC depletion alone or associated with PD did not alter this parameter. SHRs showed a more severe alveolar bone loss compared to W, and MC depletion significantly inhibited this response in both strains, with a more significant response in SHRs. MCs were less abundant in 48/80+PD groups, thus validating the previous MCs depletion in our model. PD increased the number of MC in the gingival tissue of SHR. Cytokine production (TNF-α, IL-6, IL-1β, and CXCL3) was constitutively higher in SHR and increased further after PD, which was also significantly reduced in the MCs-depleted animals. PD led to an increased expression of *Opn*, *Rankl*, *Rank*, *Vtn*, *Itga5*, *Itgb5*, *Trap*, and *Ctsk* in the mandible of W and SHRs, which was reversed in MCs-depleted animals. These results suggest that MCs significantly contributes to the PD-induced alveolar bone resorption, especially in the SHR, which is associated with a more severe PD progression compared to Wistar, partly explained by these cells contribution to the inflammatory status and mediator production, stimulating osteoclast-related response markers, which were reduced after MC depletion in our experimental model.

## Introduction

Periodontal disease (PD) is an inflammatory disorder of the tissue surrounding and supporting the teeth, with alveolar bone resorption and teeth-loss as the most severe presentations [1]. The disorder is initiated by dental biofilm accumulation caused by bad oral care and is a public health issue worldwide [2]. Although the disease progresses from a dynamic host-pathogen interaction, depending on the microbial pathogenesis, an important concern is that it may be aggravated by an exacerbated host immune response, like increased production of inflammatory mediators [3, 4].

Various systemic conditions, like hypertension, may influence PD progression. These conditions share many risk factors and are likely to coexist in many individuals [5]. Hypertension increases the systemic inflammatory burden and may alter the response of the non-immune host cells, like fibroblasts, epithelial cells, and recruited leukocytes, and also resident immune cells, like macrophages and MCs [6, 7].

Mechanisms related to MCs induced inflammation are still not well understood, but they act as the key effectors in tissue injury responses. They are mostly localized to blood vessels surrounding the subcutaneous and mucous tissues and are well known for a wide spectrum of stored preformed inflammatory mediators that are released upon stimulus [8]. Carranza and Cabrini [9] demonstrated the presence of MCs in the periodontium and showed that gingivitis severity correlates to increasing MCs numbers. Many studies have since reported evidence supporting the significant role of MCs in inflammation-driven soft and hard tissue disruption [10, 11]. However, despite sufficient knowledge on PD pathogenesis, the MCs-related mechanisms are still not well understood.

The Spontaneously Hypertensive Rat (SHR) is a widely used animal model that shares several characteristics with the physiology of human hypertension, like being multifactorial and genetically determined [12]. It has also been shown that the SHR presents intrinsic bone alterations and increased inflammatory status, which are related to the hypertensive genotype and phenotype [13–15]. Furthermore, evidence has shown a relationship between hypertensive conditions and a possible increased mast cell activity, mediated by mechanisms yet to be elucidated [16, 17].

Compound 48/80 is an MC secretagogue agent, experimentally used to promote MC degranulation, and has been used as an *in vivo* MC-depleting agent [18–20]. Therefore, this model was chosen for our present study, in which we focused on the role of MCs in PD-induced bone alterations in hypertensive rats.

Our data corroborate with a more severe PD-induced alveolar bone loss in the SHR, accompanied by increased production of inflammatory mediators and increased osteoclasts-related mechanisms. Previously, these characteristics were significantly inhibited in rat mandibles upon MC depletion and showed a more pronounced contribution of these cells in hypertensive strains.

## Materials and methods

### Animals and experimental groups

A total of 66 age-matched (10-week-old) male rats from Wistar and SHR strains (*Rattus norvegicus albinus*) from the Central Animal Facility of School of Dentistry of Araçatuba (Unesp) were used. Animals were housed in a controlled light, temperature, and humidity environment (12h light/dark cycle, 22±2°C, and 55±5%), and offered a standard pellet diet and drinking water ad libitum. Animals from each strain (Wistar, W; and SHR, S) were randomly divided into the following experimental groups: Control (C), animals with PD (PD), and mast cell-depleted animals with PD (48/80+PD). Additionally, for a mast-cell depleted control group (without PD; W+48/80 and S+48/80) six male Wistar and six male SHR without PD were used. Experimental protocols were in accordance with Brazil’s National Council for Animal Experiments Control and were approved by the Ethics Committee on Animal Use from the School of Dentistry of Araçatuba (Unesp) (Process FOA-00686-2016).

### Mast cells depletion

Mast cells depletion was conducted by the mast cell degranulation-inducing drug, compound 48/80 (c48/80; Sigma-Aldrich; St. Louis, Missouri, USA) [21, 22]. Briefly, c48/80 was administered intraperitoneally twice a day for four days, with increasing doses (day 1: 0.6; day 2 and 3: 1.2; and day 4: 2.4 mg/Kg), so that on day 5, periodontal disease was induced in the absence of filled-vesicles mast cells. Non-treated animals only received the vehicle (sterile phosphate-buffered saline, PBS).

### Periodontal disease (PD) induction

PD was induced by bilateral ligature inserted around the lower first molars and kept for 15 days, as previously described [23]. Briefly, animals were anesthetized (ketamine and xylazine hydrochloride association, 80 and 10 mg/Kg) and placed in ventral decubitus on a dental table for rodents, with oral retractors supported on incisive teeth. A 4–0 silk thread (Shalon; Goiânia, Goiás, Brazil) was wrapped around the first inferior molars, carefully pushed into the gingival sulcus, and knotted medially.

### Non-invasive blood pressure measurement

To confirm the hypertensive phenotype systolic blood pressure (SBP) was verified by tail plethysmography (NIBP and PowerLab System; ADInstruments; Sydney, Australia) after the 15 days of PD, pre-treated or not with c48/80, and animals were considered hypertensive when SBP ≥150 mmHg [24].

### Euthanasia and sample harvest

On day 15 after PD induction, animals were euthanized by inhalational anesthetic overdose (Isoflurane, Cristália, Itapina, SP, Brazil), the presence of bilateral ligature was evaluated, and animals in which it was absent were excluded from the study. The hemi-mandibles and gingival tissue were surgically collected, and the right-sided bones were stored in PBS at -20°C for microtomography, or fixed in 10% buffered formaldehyde for histological processing, and left-sided bones were immediately frozen in liquid nitrogen and stored at -80°C, for gene expression analysis and cytokines quantitation.

### Gingival mast cell count

Formaldehyde fixed gingiva was paraffin-embedded and 3-μm thick sagittal sections were obtained and used for toluidine blue staining. For mast cell counting, representative slides were photographed, the connective gingival tissue area was determined, and the mast cell number (purpled-stained cells) were counted (n = 5/group), using the ImageJ software (v1.47, National Institutes of Health).

### Micro-computed tomography (microCT) analysis

Tomographic images from left hemi-mandibles (n = 5/group) were acquired using a SkyScan 1272 system (Bruker microCT; Kontich, Belgium) [70 kVp and 142 μA; 0.5 mm aluminum filter; 9 μm isotropic voxel; 1100 ms exposure time, 2 frames averaging, and 180° rotation (0.5° rotation step)], and three-dimensionally reconstructed (NRecon software; v1.6; Bruker microCT). Alveolar bone loss was evaluated in the first molar region, a region of interest (ROI) was standardized from defined anatomical points (upper limit: furcation roof; lower limit: proximal root apex; distal limit: 2nd molar proximal root; proximal limit: 1st molar proximal root, and vestibular and lingual limits: limits of the alveolar bone). A volume of interest (VOI) was automatically delimited by the bone edges and the tooth volume exclusion. The bone percentage (BV/TV), trabecular number (Tb.N), thickness (Tb.Th), and separation (Tb.Sp) were analyzed [25].

### Cytokine quantification

The left hemi-mandibles (n = 6/group) were sectioned from the mandibular branch and incisor teeth, powdered in liquid nitrogen, and subsequently homogenized with a lysis buffer (100 mM Tris-HCl, 150 mM NaCl, 1% Tween 20, 0.5% sodium deoxycholate, 1 mM EDTA, pH 7.4). The samples were centrifuged and in the recovered supernatant, TNF-α, IL-6, IL-1β, CXCL3/CINC-2, and CCL20/MIP-3α were quantified by enzyme-linked immunosorbent assay (ELISA), with DuoSet® ELISA (in order: DY510, DY506, DY501, DY540, and DY516; R&D Systems, Minneapolis, MN, USA). Results were normalized by the total protein content, determined by the Lowry method [26].

### Gene expression analysis

Left hemi-mandibles (n = 6/group) were sectioned, excluding the mandibular branch and the incisor tooth, powdered in liquid nitrogen, and total RNA was extracted using TRIzol reagent (Invitrogen, Thermo Fisher Scientific; Carlsbad, CA, USA) following the manufacturer’s instructions. RNA purity was assessed by 260/280 and 260/230 spectrophotometry ratio (satisfactory between 1.8–2.0, and 2.0–2.2, respectively). Samples were treated with DNAse I (Sigma-Aldrich), RNA was quantified (Quant-iT RiboGreen RNA Assay Kit, Invitrogen) and 2 μg of total RNA were reverse transcribed to complementary DNA (High Capacity RNA-to-cDNA™ Kit; Applied Biosystems, ThemoFisher Scientific; Foster City, California, USA), according to manufacturer’s instructions.

Gene expression analysis of bone markers and cytokines was performed by qRT-PCR, in StepOne PlusTM Real-Time PCR Systems, with TaqMan^TM^ Gene Expression Assays (FAM fluorophore reporter / non-fluorescent quencher MGB) (Applied Biosystems, Thermo Fisher Scientific). Targets expression was normalized by *Actb* expression, as the housekeeping gene, and relative transcripts abundance was determined by the 2^(-delta Ct)^ method [27]. Assay references are listed in Table 1.

**Table 1:** Taqman® assay reference list for qRT-PCR.

**Transcription factors**		
*Runx2*	Runt-related transcription factor 2	*Rn01512298_m1*
*Osx/Sp7*	Osterix/Sp7 transcription factor	*Rn02769744_s1*
*Ctnnb*	β-catenin/Cadherin associated protein beta 1	*Rn00584431_g1*
**Bone formation markers**		
*Alp*	Bone alkaline phosphatase	*Rn01516028_m1*
*Col1a1*	Collagen type I alpha 1	*Rn01463848_m1*
*Opn/Spp1*	Osteopontin/secreted phosphoprotein 1	*Rn00681031_m1*
*Ocn/Bglap*	Osteocalcin/Bone gamma-carboxyglutamate protein	*Rn00566386_g1*
*Bsp/Ibsp*	Bone sialoprotein/Integrin-binding sialoprotein	*Rn00561414_m1*
*Bmp2*	Bone morphogenetic protein 2	*Rn00567818_m1*
**Bone formation/remodeling markers**		
*Opg*	Tumor necrosis factor receptor superfamily member 11b	*Rn00563499_m1*
*Rankl*	Tumor necrosis factor ligand superfamily member 11	*Rn00589289_m1*
*Rank*	Tumor necrosis factor receptor superfamily member 11a	*Rn04340164_m1*
*Oscar*	Osteoclast associated immunoglobulin-like receptor	*Rn01530958_m1*
*Vtn*	Vitronectin	*Rn01466920_g1*
*Itgav*	Integrin, alpha V	*Rn01485633_m1*
*Itgb5*	Integrin, beta 5	*Rn01439348_m1*
*Mmp2*	Matrix metalloproteinase 2	*Rn01538170_m1*
*Mmp9*	Matrix metalloproteinase 9	*Rn00579162_m1*
*Trap/Acp5*	Acid phosphatase 5, tartrate resistant	*Rn00569608_m1*
*Ctsk*	Cathepsin K	*Rn00580723_m1*
**Cytokines**		
*IL-1β*	Interleukin-1β	*Rn00580432_m1*
*TNF-α*	Tumor necrosis factor	*Rn01525859_g1*
*IL-6*	Interleukin-6	*Rn01410330_m1*
**Housekeeping gene**		
*Actb*	β-actin	*Rn00667869_m1*

### Immunohistochemistry assays

Formaldehyde fixed mandibles (n = 5/group) were decalcified in 10% EDTA-buffered solution (Titriplex® III; Merck Millipore; Burlington, MA, USA), for paraffin histological processing. Mandibles 3-μm thick sagittal sections were obtained and used for hematoxylin and eosin staining, and immunostaining of bone markers. Briefly, tissue sections were deparaffinized, rehydrated, and submitted to endogenous peroxidase blocking, and hot citric acid buffer antigen retrieval. immunostaining was performed by primary antibody incubation (references listed in Table 2) and detected by Histofine® Simple Stain™ kit (Nichirei Biosciences Inc.; Tokyo, Japan), followed by the chromogenic substrate (3,3’-diaminobenzidine-tetrahydrochloride; Dako Corp., Carpinteria, CA, USA), according to manufactures instruction. Harry’s hematoxylin was used as counterstaining, and all assays were accompanied by a negative control slide which was submitted to the same procedures, except for the primary antibody incubation.

**Table 2:** Antibody reference list for immunohistochemistry assays.

Primary antibody	Source	Reference	Manufacturer
Anti-RUNX2 (F-2)	Mouse	sc-390351	Santa Cruz Biotechnology.
Anti-OSTERIX (Y-21)	Rabbit	sc-133871	Santa Cruz Biotechnology
Anti-CTNNB (12F7)	Mouse	sc-59737	Santa Cruz Biotechnology
Anti-COL1A1 (L-19)	Goat	sc-8783	Santa Cruz Biotechnology
Anti-OPN	Rabbit	ab8448	Abcam
Anti-OPG (N-20)	Goat	sc-8468	Santa Cruz Biotechnology
Anti-RANKL (N-19)	Goat	sc-7628	Santa Cruz Biotechnology
Anti-RANK (H-300)	Rabbit	sc-9072	Santa Cruz Biotechnology
Anti-VTN (EP873Y)	Rabbit	ab45139	Abcam
Anti-ITGαVβ3 (23C6)	Mouse	sc-7312	Santa Cruz Biotechnology
Anti-MMP2 (2C1)	Goat	sc-13594	Santa Cruz Biotechnology
Anti-MMP9 (C-28)	Goat	sc-6841	Santa Cruz Biotechnology
Anti-TRAP (N-17)	Goat	sc-30832	Santa Cruz Biotechnology
Anti-CTSK (N-20)	Goat	sc-6507	Santa Cruz Biotechnology
Anti-BMP2	Rabbit	ab1493	Abcam
Anti-OCN (FL-95)	Rabbit	sc-30045	Santa Cruz Biotechnology
Anti-BSP (M-154)	Rabbit	sc-292394	Santa Cruz Biotechnology

For IHC analysis, slides were blindly analyzed (n = 5/group) in 400X magnification bright-field microscopy in the region of interest (middle third of the first molar furcation region), and expression was based on the immunostaining pattern [negative (−); low staining (+); moderate staining (++); strong staining (+++)] [28]. Image boards present a photographed representative section, after digital correction for brightness and contrast, and black arrows indicate positive-stained cells.

### Statistical analysis

Data are expressed as mean and standard error of the mean (SEM), and were analyzed by one-way ANOVA, followed by Šidak *post hoc* test for multiple comparisons, after being tested for normality distribution by Shapiro-Wilk test. Statistical difference is represented by brackets labelled by * p<0.05, ** p<0.01, *** p<0.001, and **** p<0.0001, comparing Control, PD and 48/80+PD, or Wistar and SHR in the same experimental condition. All analysis was performed on Graph Pad Prism v8.0 (GraphPad Software Inc.; San Diego, California, USA).

## Results

### Previous mast cells depletion does not alter blood pressure after PD

To ensure the SHR phenotype, we evaluated the systolic blood pressure (SBP) after 15 days of PD. As expected, all SHR groups presented elevated SBP, compared to W groups (Table 3), but previous mast cell depletion alone or associated with PD did not alter this parameter.

**Table 3:** Systolic Blood Pressure (SBP) of Wistar and SHR with PD, depleted from mast cells (48/80+PD).

	SBP		
	Mean (mmHg)	SEM	*n*
WC	102,40^**a**^	3,20	6
W+48/80 *(without PD)*	104,70 ^**a**^	4,08	6
WPD	116,30 ^**a**^	5,03	6
W48/80+PD	109,80 ^**a**^	3,31	6
SC	162,30 ^**b**^	2,58	6
S+48/80*(without PD)*	159,50 ^**b**^	3,95	6
SPD	157,90 ^**b**^	3,20	6
S48/80+PD	157,80^**b**^	1,18	6

SBP measured by tail plethysmography are shown as mean and SEM (n = 6). Significant statistical differences are represented by a different superscript letter (^a^ and ^b^; p<0.001).

### Mast cells are more abundant in SHR inflamed gingival tissue

**Fig 1:**
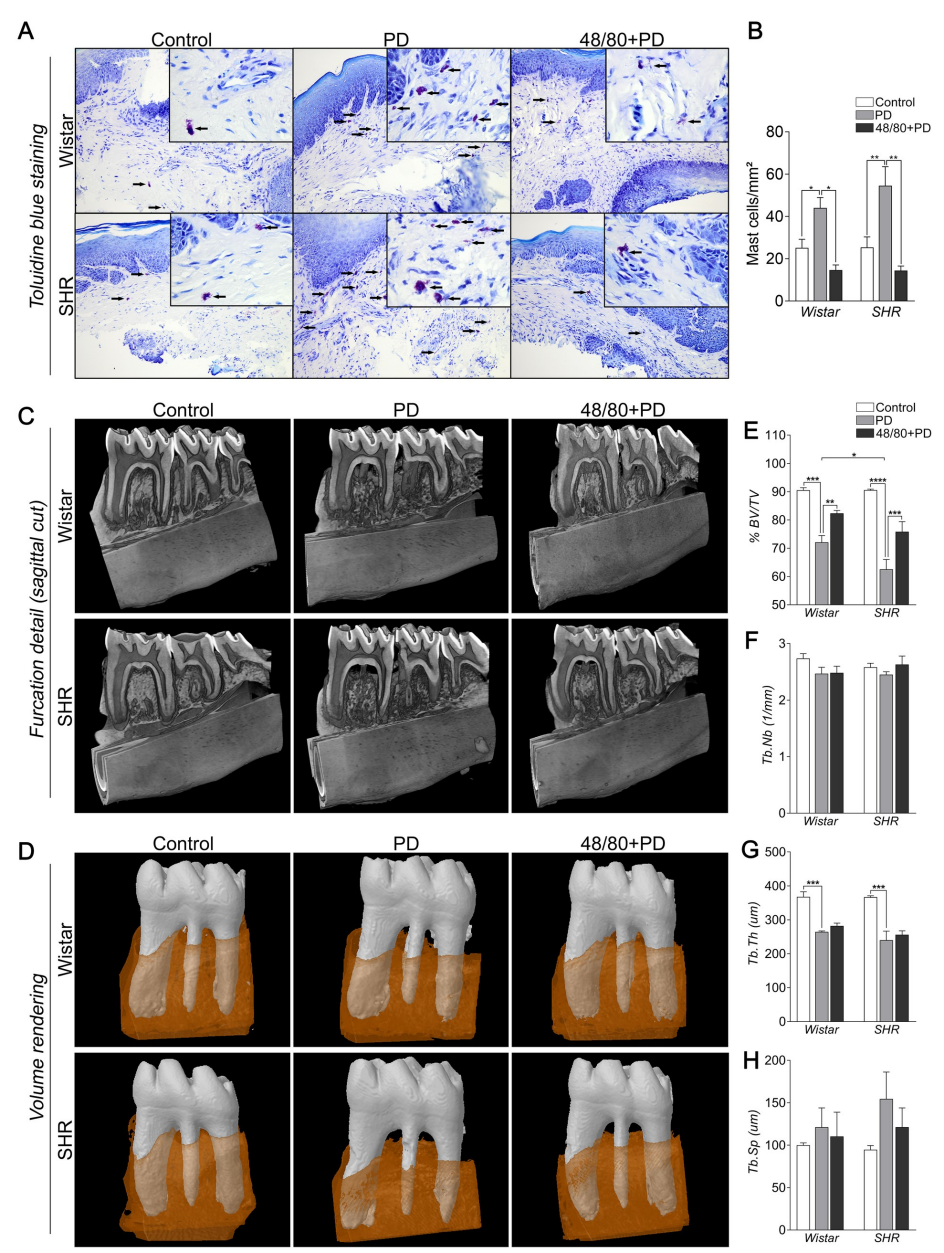
Mast cell count in the gingival tissue of Wistar and SHR with PD, depleted from mast cells (48/80+PD). (A) Representative images of toluidine blue stained gingival section. The black arrows indicate mast cells (purple-stained cells). (B) Graph shows mean ± SEM (n = 5) of mast cell count per mm^2^, and statistical differences are represented by brackets labeled by *p<0.05, **p<0.01, ***p<0.001, and ****p<0.0001. **Microtomography analysis of mandible of Wistar and SHR with PD, depleted from mast cells (48/80+PD).** (C) Mandible three-dimensional reconstruction in lingual view with sagittal cut detail, (D) Volume rendering of the analyzed region (first molar teeth in white and surrounding alveolar bone in brown), and graphs showing (E) %BV/TV, (F) Tb.Nb, (G) Tb.Th, and (H) Tb.Sp as mean ± SEM (n = 5). Statistical differences are represented by brackets labeled by * p<0.05, ** p<0.01, *** p<0.001, and **** p<0.0001.

The presence of MCs in the gingival tissue was verified by toluidine blue staining (Fig 1A and 1B). Few MCs were observed in the control groups (WC and SC). However, they were more abundant in groups with PD, especially in the SPD group, suggesting participation in PD inflammatory responses. As expected, MCs were less abundant in 48/80+PD groups, thus validating the previous MCs depletion in our model.

### Mast cells depletion prevented PD-induced alveolar bone loss in SHR

We analyzed the alveolar bone loss and bone architecture parameter by microCT (Fig 1C–1H). As expected, WPD and SPD groups showed significant bone loss with lower %BV/TV and Tb.Th, compared to their respective controls (Fig 1E and 1G), while Tb.Nb and Tb.Sp were not significantly altered (Fig 1F and 1H). Although WC and SC had similar alveolar bone structures (Fig 1C–1E), bone loss was more severe in SPD, as compared to WPD. Interestingly, MCs depletion was able to prevent this loss, more significantly in S48/80+PD, but the trabecular architecture was not significantly altered (Fig 1F–1H). We also evaluated if previous MC depletion alone would alternate the alveolar bone loss, which was not noticed (S1 Fig).

### Mast cells depletion reduced PD-induced inflammation in SHR

**Fig 2:**
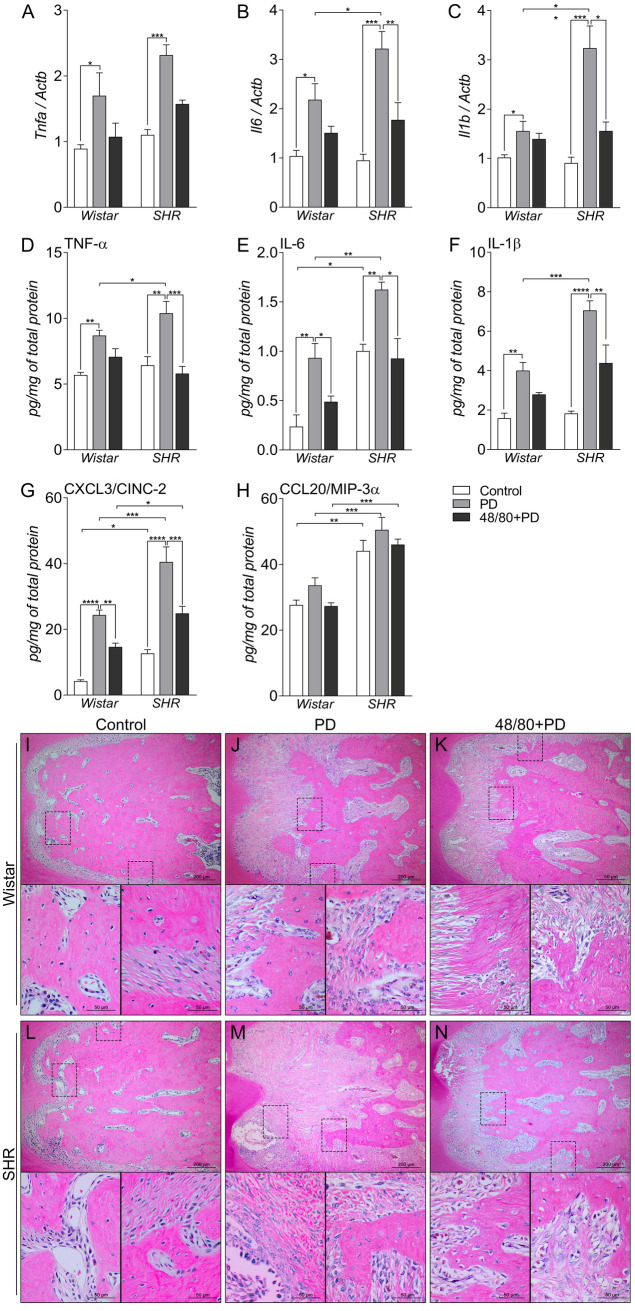
Inflammatory status in mandibles of Wistar and SHR with PD 15d, depleted of mast cells (48/80+PD). qRT-PCR for (A) Tnfa, (B) Il6, and (C) Il1b, and ELISA for (D) TNF-α, (E) IL-6, (F) IL-1β, (G) CXCL3 and (H) CCL20. Graphs shown mean ± SEM (n = 6), and statistical difference are represented by brackets labeled by * p<0.05, ** p<0.01, *** p<0.001, and **** p<0.0001. (I-N) H&E staining of the first mandibular molar, in the furcation region, image board shows representative images from each experimental group (n = 5).

To assess the inflammatory process, we evaluated the gene expression of *Tnfa*, *Il6*, and *Il1b* by qRT-PCR, and the production of TNF-α, IL-6, IL-1β, CXCL3/CINC-2, and CCL20/MIP-3α by ELISA in the rat mandibles (Fig 2A–2H). *Tnfa*, *Il6*, and *Il1b* expression were higher in PD groups, compared to their respective controls (Fig 2A–2C). MCs depletion only reduced the expression of *Il6* and *Il1b* in S48/80+PD group, compared to SPD (Fig 2B and 2C). When quantified at the protein level, TNF-α, IL-6, and IL-1β were also increased in WPD and SPD compared to their controls, and were significantly higher in SPD, compared to WPD (Fig 2D–2F). In S48/80+PD, all mediators were reduced, compared to SPD, while in W48/80+PD only IL-6 was reduced, compared to WC (Fig 2D–2F).

The chemotactic proteins CXCL3/CINC-2 and CCL20/MIP-3α were also quantified, which are associated with neutrophil and macrophage infiltrate, respectively. CCL20 was not altered in PD, but had inherently higher levels in SHR groups, compared to Wistar groups (Fig 2H). CXCL3, however, was significantly increased in PD groups but was partially prevented in 48/80+PD groups.

H&E staining corroborated and further demonstrated the periodontal inflammation response (Fig 2I–2N). Histological observation showed increased leukocyte infiltration and periodontal ligament disorganization to be more severe in SPD (Fig 2M), compared to WPD (Fig 2J), which was prevented in 48/80+PD groups (Fig 2K and 2N).

### Mast cells depletion reduced *Ctnnb* expression

**Fig 3:**
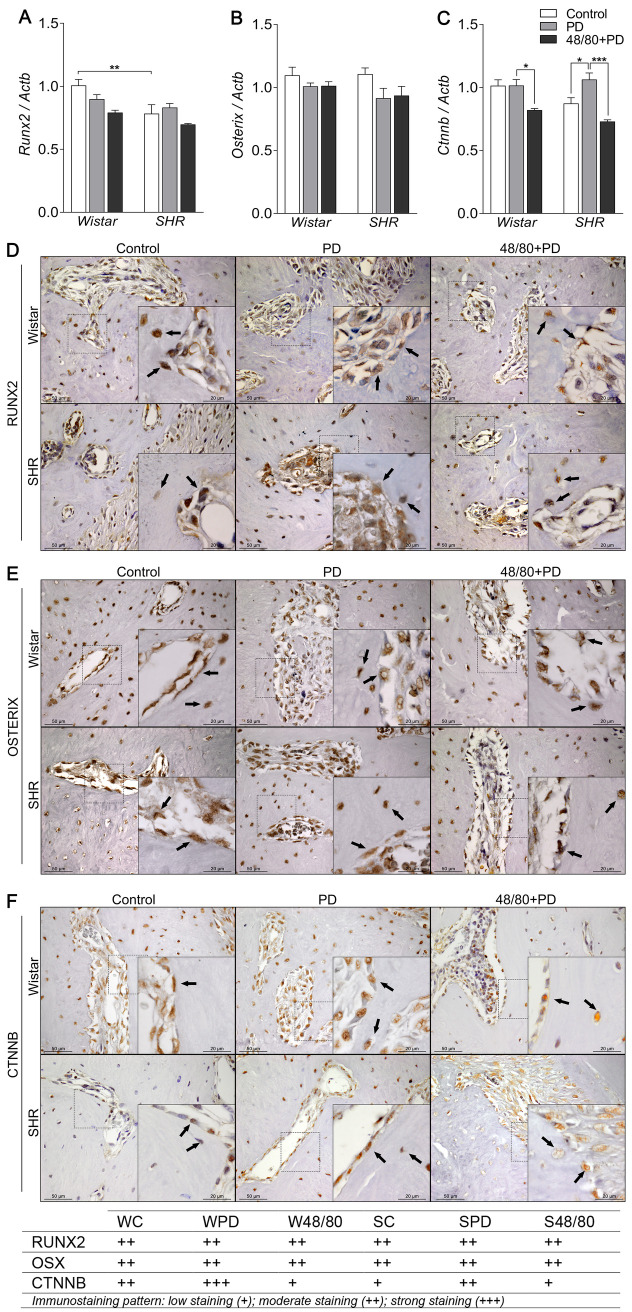
Transcription factors expression in mandibles of Wistar and SHR with PD, depleted of mast cells (48/80+PD). qRT-PCR for (A) Runx2, (B) Osterix, (C) Catnb, (D) immunostaining for (D) RUNX2, (E) OSTERIX, and (F) CTNNB. Graphs shown mean ± SEM (n = 6), and statistical difference are represented by brackets labeled by * p<0.05, ** p<0.01, *** p<0.001, and **** p<0.0001. The image boards show representative images from each experimental group (n = 5), and tables describe the average immunostained pattern of each target.

To study the mechanisms modulated by MCs in the PD-associated alveolar bone loss, we analyzed the expression of the main bone markers in the rat mandibles by qRT-PCR and immunostaining. The transcription factors *Runx2*, *Osx*, and *Ctnnb* were initially analyzed (Fig 3A–3C). SC had a lower *Runx2* expression compared to WC (Fig 3A), while *Osx* and *Ctnnb* expression were similar in both groups (Fig 3B and 3C). PD led to an increased *Ctnnb* expression in SPD only. However, MCs depletion was able to significantly reduce its expression in WPD and S48/80+PD (Fig 3C). Immunostaining confirmed this response (Fig 3F) as CTNNB showed no alteration in WPD compared to WC, but weaker staining in the W48/80+PD, while it was increased in SPD as compared to SC, which was partially prevented in S48/80+PD (Fig 3D). RUNX2 and OSX labeling did not show significant differences between any groups (Fig 3D and 3E).

### Mast cells modulate *Opn* expression in the alveolar bone

**Fig 4:**
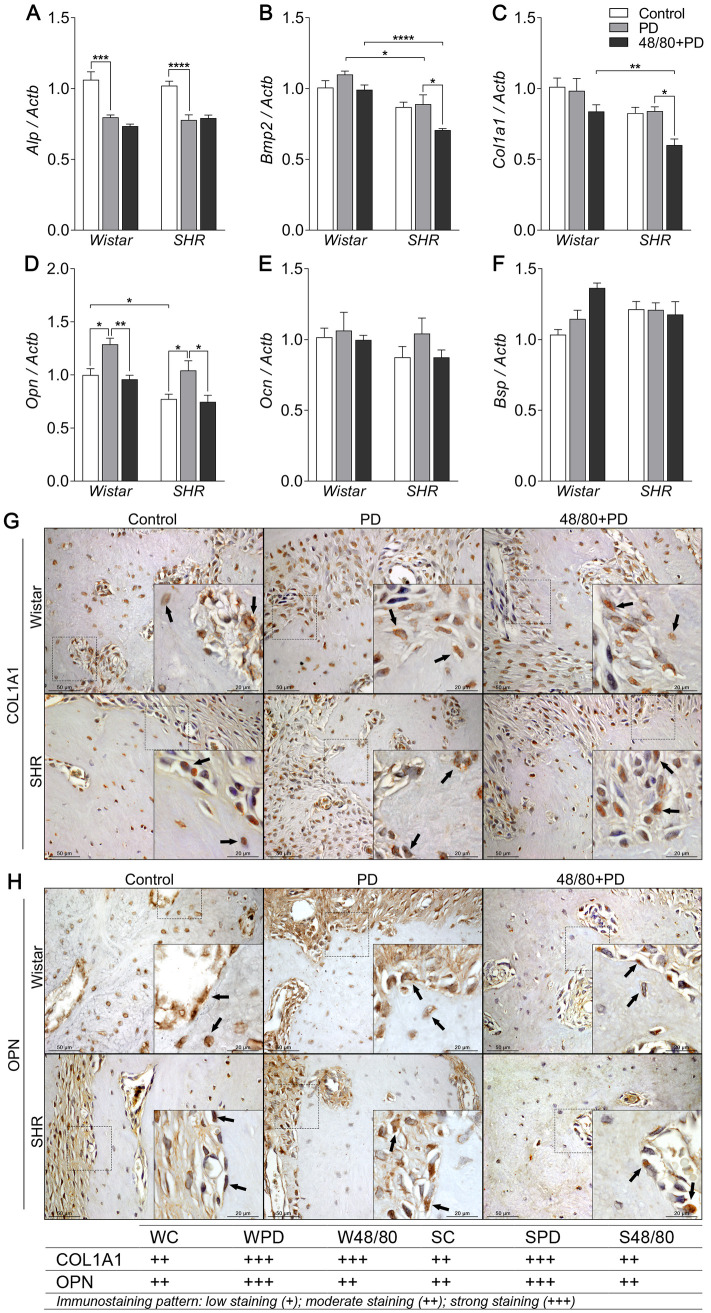
Bone formation markers expression in mandibles of Wistar and SHR with PD, depleted of mast cells (48/80+PD). qRT-PCR for (A) Alp, (B) Bmp2, (C) Col1a1, (D) Opn, (E) Ocn, and (F) Bsp, and immunostaining for (G) COL1A1, and (H) OPN. Graphs shown mean ± SEM (n = 6), and statistical difference are represented by brackets labeled by * p<0.05, ** p<0.01, *** p<0.001, and **** p<0.0001. The image boards show representative images from each experimental group (n = 5), and tables describe the average immunostained pattern of each target.

We then analyzed the expression of bone formation markers *Alp*, *Bmp2*, *Col1a1*, *Opn*, *Ocn*, and *Bsp* (Fig 4A–4F). We observed a significant decrease in *Alp* expression in PD groups compared to control groups, but MCs depletion did not modulate this response (Fig 4A). *Bmp2*, *Col1a1*, *Ocn*, and *Bsp* were not altered in PD (Fig 4B, 4C and 4F). However, the S48/80+PD group showed a decreased expression of *Bmp2* and *Col1a1* (Fig 4B and 4C). IHC analysis did not show significant differences in the BMP2 staining (S2 Fig), however, COL1A1 staining was increased in PD groups, but only reduced in S48/80+PD, compared to SPD (Fig 4G).

SC showed an inherently lower *Opn* expression compared to WC, but PD led to an increased expression in WPD and SPD, compared to the respective controls (Fig 4D). Interestingly, MCs depletion significantly reduced *Opn* expression in WPD and S48/80+PD (Fig 4D). IHC analysis confirmed the OPN response, by and increased staining in WPD compared to WC, mostly seen in the bone adjacent connective tissue, while it was reduced in W48/80+PD in both the connective tissue and bone cells (Fig 4H). SC presented a constitutive higher OPN staining, which was further increased in bone cells in the SPD group, but reduced in S48/80+PD (Fig 4H).

### Mast cells depletion prevented altered *Opg/Rankl/Rank* axis expression

**Fig 5:**
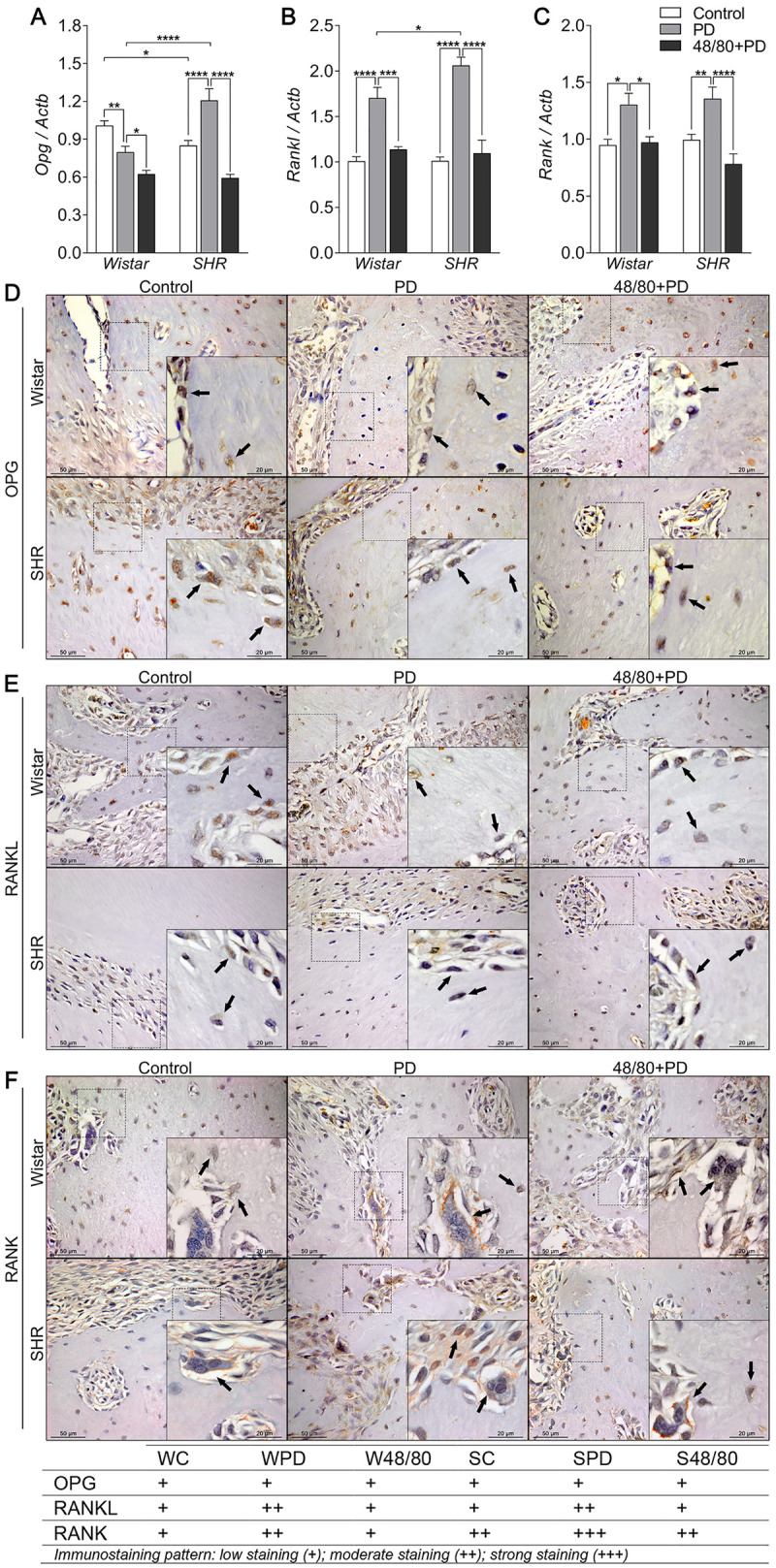
Opg, Rankl and Rank axis in mandibles of Wistar and SHR with PD, depleted of mast cells (48/80+PD). qRT-PCR for (A) Opg, (B) Rankl, (C) Rank, and immunostaining for (D) OPG, (E) RANKL, and (F) RANK. Graphs shown mean ± SEM (n = 6), and statistical difference are represented by brackets labeled by * p<0.05, ** p<0.01, *** p<0.001, and **** p<0.0001. The image boards show representative images from each experimental group (n = 5), and tables describe the average immunostained pattern of each target.

We also evaluated the *Opg/Rankl/Rank* axis expression in rat mandibles (Fig 5), which plays a major role in bone remodeling and resorption. We noticed a constitutive lower *Opg* expression in SC compared to WC, but *Rankl* and *Rank* expression was similar (Fig 5A–5C). In Wistar rats, WPD showed a reduced *Opg* and increased *Rankl* and *Rank* expression. MCs depletion further decreased *Opg* expression and prevented increases in *Rankl* and *Rank* expression (Fig 5A–5C). Conversely in SHR, SPD groups had increased expression of all three axis components, which were reduced in the MCs-depleted group (Fig 5A–5C).

Immunostaining was performed to better observe this response (Fig 5D–5F). WPD showed weaker OPG staining in bone cells compared to WC, which was partially prevented in the W48/80+PD (Fig 5D). Interestingly, SC had constitutive higher OPG staining in the bone adjacent connective tissue, also observed in the SPD, but with weaker OPG staining in the bone cells (Fig 5D), and average weaker staining was observed in S48/80+PD (Fig 5D). RANKL, in turn, had a weak immunostaining pattern. However, increased staining was noticed in the PD group bone cells and adjacent connective tissues, which was slightly reduced in 48/80+PD groups (Fig 5E). Interestingly, RANK had an average intenser staining in SHR groups. In the PD groups, an increased staining was observed compared to the respective control groups, which was partially prevented in the 48/80+PD groups (Fig 5F).

### Mast cells depletion prevented an increase in resorption markers

**Fig 6:**
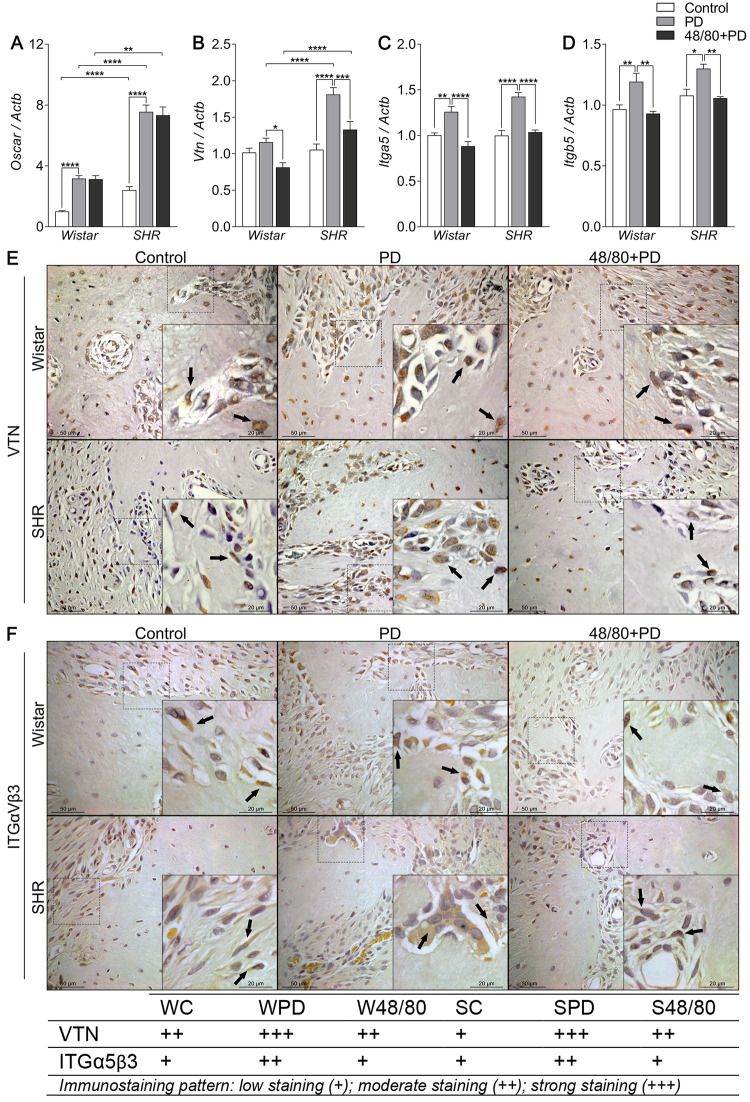
Osteoclast recruitment and activation markers expression in mandibles of Wistar and SHR with PD, depleted of mast cells (48/80+PD). qRT-PCR for (A) Oscar, (B) Vtn, (C) Itga5, (D) Itgb5, and immunostaining for (E) VTN, and (F) ITGαVβ3. Graphs shown mean ± SEM (n = 6), and statistical difference are represented by brackets labeled by * p<0.05, ** p<0.01, *** p<0.001, and **** p<0.0001. The image boards show representative images from each experimental group (n = 5), and tables describe the average immunostained pattern of each target.

**Fig 7:**
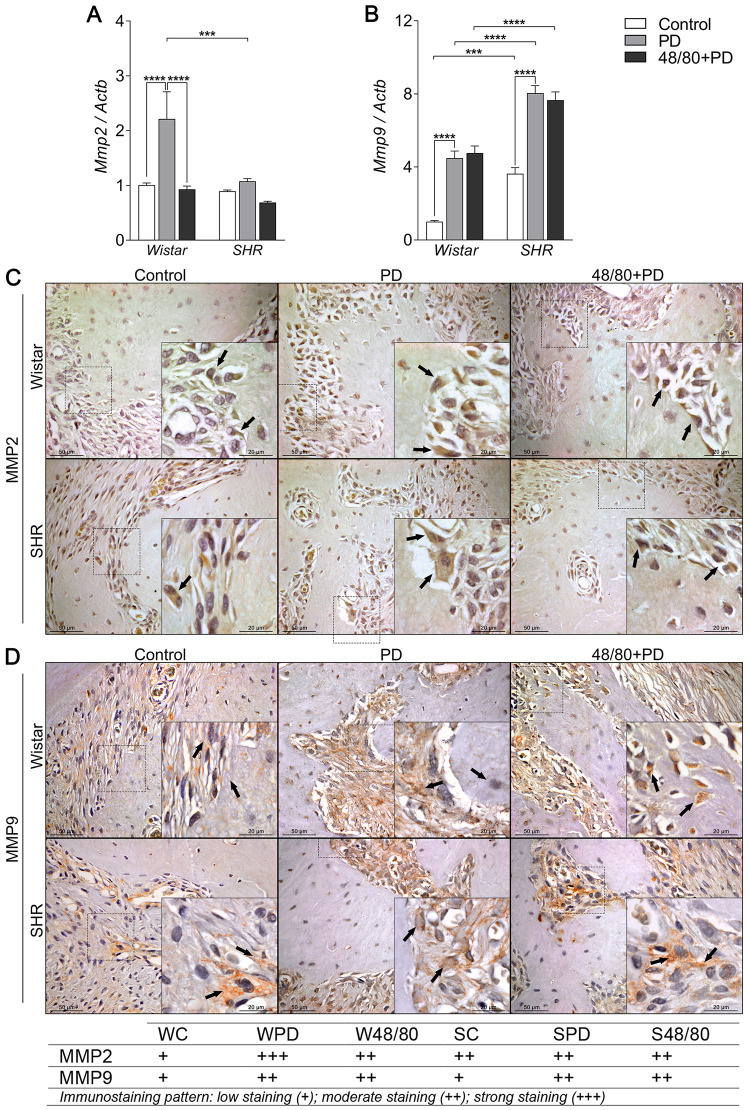
Organic matrix degradation markers expression in mandibles of Wistar and SHR with PD, depleted of mast cells (48/80+PD). qRT-PCR for (A) Mmp2 and (B) Mmp9, and immunostaining for (C) MMP2, and (D) MMP9. Graphs shown mean ± SEM (n = 6), and statistical difference are represented by brackets labeled by * p<0.05, ** p<0.01, *** p<0.001, and **** p<0.0001. The image boards show representative images from each experimental group (n = 5), and tables describe the average immunostained pattern of each target.

**Fig 8:**
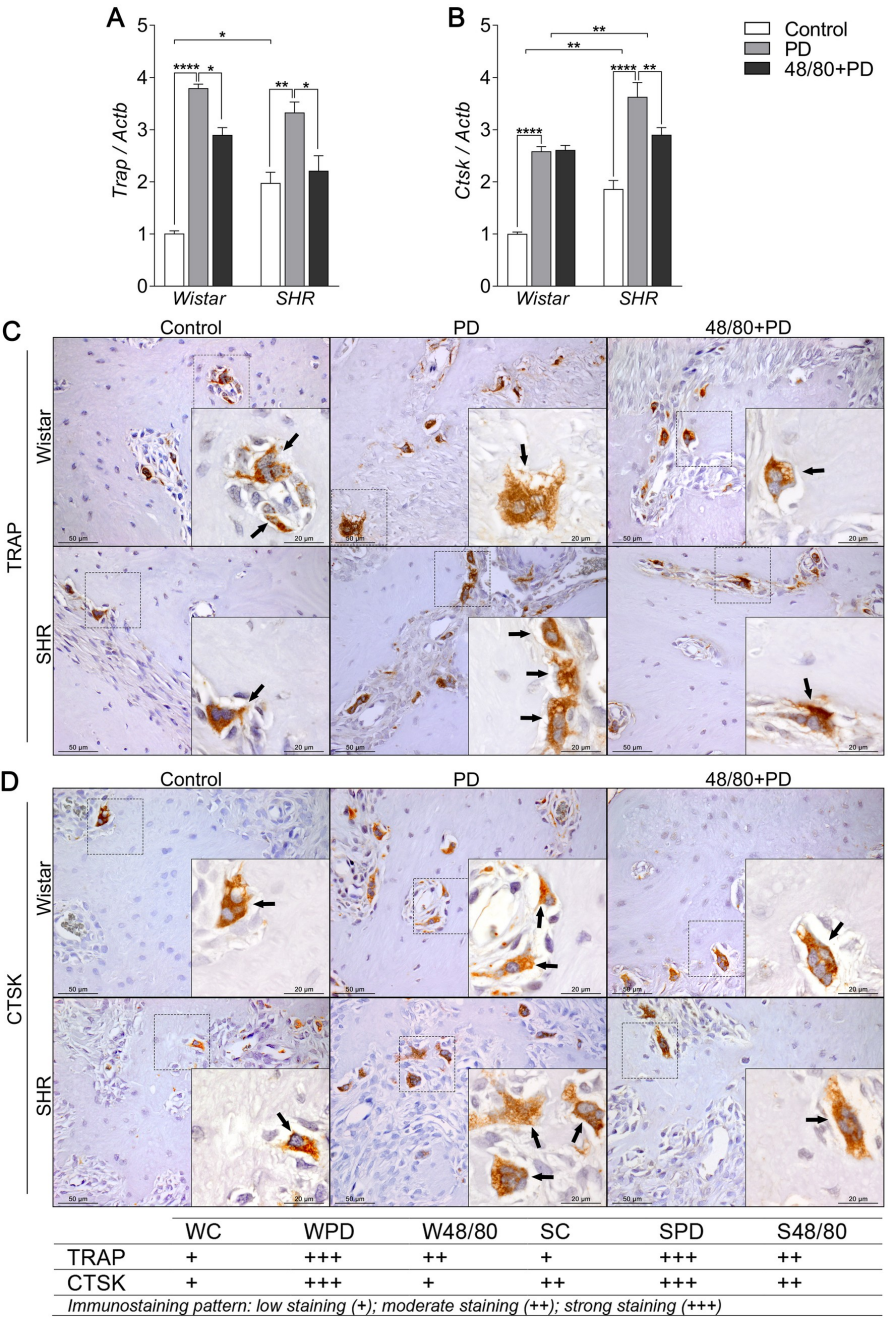
Osteoclast function markers expression in mandibles of Wistar and SHR with PD, depleted of mast cells (48/80+PD). qRT-PCR for (A) Trap and (B) Ctsk, and immunostaining for (C) TRAP, and (D) CTSK. Graphs shown mean ± SEM (n = 6), and statistical difference are represented by brackets labeled by * p<0.05, ** p<0.01, *** p<0.001, and **** p<0.0001. The image boards show representative images from each experimental group (n = 5), and tables describe the average immunostained pattern of each target.

Finally, we evaluated the markers associated with bone resorption, including markers of osteoclast recruitment and activation, namely *Oscar*, *Vtn*, *Itga5*, and *Itgb5* (Fig 6A–6F); organic matrix degradation, *Mmp2* and *Mmp9* (Fig 7A and 7D); and osteoclast function *Trap* and *Ctsk* (Fig 8A–8D).

The results showed SC to have a higher constitutive expression of *Oscar*, *Mmp9*, *Trap*, and *Ctsk*, compared to WC (Figs 6A, 7A, 8A and 8B). In WPD, there was an increased expression of all analyzed markers, compared to WC, except for *Vtn* (Fig 6B). We observed a reduced *Vtn*, *Itga5*, *Itgb5*, *Mmp2*, *and Trap* expression in W48/80+PD, compared to WPD (Figs 6B–6D and 7A). SHR showed a slightly different response, where PD increased the expression of all analyzed targets, except for *Mmp2* (Fig 7A), while in S48/80+PD there was a reduced expression of *Vtn*, *Itga5*, *Itgb5*, *Trap*, and *Ctsk* (Figs 6B–6D, 8A and 8B).

To further confirm these tissue responses in the previous MCs-depleted animals, we evaluated the immunostaining of the main altered markers. Immunostaining confirmed the response patter of VTN and ITG, showing an increased staining in the PD groups, while reduced in 48/80+PD. However, ITG staining alterations were concentrated in the bone cells, while VTN was equally distributed in bone cells and the adjacent connective tissue (Fig 6E and 6F).

MMP2 staining patterns alteration were more evident in the bone cells, while for MMP9 it was in the adjacent connective tissue. Different from gene expression analysis, MMP2 showed to be slightly higher in SC, compared to WC, while MMP9 showed a similar staining intensity between the control groups. Interestingly, IHC confirmed a MMP2 reduction in W48/80+PD, but MMP9 showed no significant changes in the 48/80+DP (Fig 7C and 7D).

Regarding the osteoclast functional markers, constitutive differences between WC and SC were not as apparent as in the gene expression of those markers. However, as expected, PD groups had more abundant TRAP- and CTSK-positive cells, especially in SPD, compared to SC and WPD. The response was prevented in W48/80+PD and S48/80+PD, which showed less stained cells compared to PD groups (Fig 8C and 8D).

## Discussion

In the present study, the MCs number was higher in inflamed gingival tissues of the SPD group, than in WPD, and also SPD presented an increased inflammatory response and severe alveolar bone loss compared to WPD, suggesting a pronounced role of MCs in the hypertensive rat.

The SHR has already been shown to have exacerbated periodontal inflammation [23, 29] and the hypertensive genotype and phenotype have been associated with intrinsic bone impairments [15, 30]. Even though we did not observe any differences in the alveolar bone microstructure between control groups, SC had an inherently lower *Opn* and *Ocn* expression, and increased expression of the resorption markers *Trap*, *Ctsk*, *Mmp2*, *Oscar*, and *Vtn*, compared to WC. This partly explains the increased susceptibility to bone destruction observed in SPD.

We then assessed the PD consequences in the alveolar bone of SHR previously depleted from MCs. Malcolm, Millington [31] reported that MCs-deficient mice (c-Kit knockout) were protected from PD-induced alveolar bone loss, which supports our data. Interestingly, the preventive effects of MCs depletion were more significant in SHR (S48/80+PD) compared to W48/80+PD, suggesting a more pronounced role of these cells in the hypertensive strain. These cells released a variety of stored, preformed mediators upon activation [32], which can directly mediate bone resorption or activate other cells to produce more mediators [33]. Additionally, the SHR has a pre-established low-grade systemic inflammatory status [34], which may contribute to a more pronounced MCs response. Although MC depletion released a lot of mediators, these were not able to alter the SHR hypertensive phenotype confirmed by the higher SBP in SHR animals even in the presence of 48/80 compound.

MCs are known to produce TNF-α, IL-6, and IL-1β, important mediators that are increased in different PD models, favoring bone resorption [35–37]. In the current study, these molecules have an increased expression, mainly in SPD, which was significantly prevented in MCs-depleted animals, especially in S48/80+PD. The histological analysis supported these findings, thereby strengthening our hypothesis. Furthermore, the alteration in CXCL3 expression in MCs-depleted animals may contribute to an important effect on leukocyte recruitment, possibly involving neutrophils, since CCL20 was not altered. Hypertension-related mechanisms, like the renin-angiotensin axis, adrenergic tonus, and electrolytes imbalance are known to be up-regulated in the SHR phenotype [38–40] and contribute to the low-grade systemic inflammation [41]. Although the exact mechanisms remain unclear, it can be speculated that mechanisms involving MCs response, favoring mediator release could be involved.

To better understand the specific effect of MCs on the bone tissue under inflammation, a panel of bone markers to highlight possible mechanisms that explain the observed responses were used. Transcription factors regulate bone metabolism by controlling the genes directly involved in bone responses [42]. We observed an increase in *Ctnnb* expression in the mandibles from SPD only. Currently, there is no evidence to suggest that MCs regulate this factor, but periodontal ligament cells isolated from inflamed tissues and stimulated by LPS have a higher *Catnb* expression, and showed an inhibitory effect on the non-canonical Wnt pathway, favoring proliferation but not osteoblast differentiation [43]. Therefore, upon the limitation of our study, it was hypothesized that a pool of inflammatory mediators might affect the expression of transcription factors.

PD groups showed a reduced expression of *Alp*. Clinical studies have correlated PD severity to higher ALP activity in the crevicular fluid and saliva, which is explained by an increased enzymatic pool derived from polymorphonuclear cells, osteoblasts, and fibroblasts in the periodontium as analyses were done by non-specific biochemical methods [44]. However, studies have shown that ALP was inhibited in human osteoblastic cells by TNF-α and IL-1β [45, 46], corroborating our data, which were also significantly increased in PD groups in the present study. Some studies show an increased *Alp* expression in the alveolar bone at later periods of alveolar bone repair, after dental extraction or orthodontic movement [45, 46]. In contrast, the lower *Alp* mRNA observed in our model may reflect the ongoing inflammatory process, in which a repair response would not take place due to the maintenance of the deleterious stimulus. Unaltered expression of *Bmp2* (an important endogenous osteoinductor) and *Col1a1* (the main bone matrix organic component) in PD groups additionally support the fact that bone formation or repair attempts are absent, and *Ocn* and *Bsp* expression, markers of bone matrix formation and maturation, were not altered either.

*Opn* was significantly elevated in PD groups, corroborating other studies that correlate an increased *Opn* in saliva, crevicular fluid, and serum, to PD progression in humans [47, 48]. Although *Opn* is present in the bone tissue, it is also related to inflammatory responses, by mediating recruitment, adhesion, and activation of immune cells [49, 50]. MCs-depleted groups had a significant reduction in *Opn* expression that could be explained by the lower inflammatory status, hinting at an important role of MCs in the modulation of cell recruitment in both strains. Conversely, decreased expression of *Bmp2* and *Col1a1* in S48/80+PD suggests a difference in the modulation of bone dynamics under inflammatory conditions, which could be further evaluated in future studies.

Concerning the aforementioned, the *Opg/Rankl/Rank* axis regulates bone remodeling and dynamics, thus maintaining its structure, mass, and strength [51]. In the present study PD induced significant increases in *Rankl* and *Rank* expression in the mandible of WPD and SPD, which can be explained by increased inflammatory mediators production [52]. *Opg* was decreased in WPD, while increased in SPD. It can be speculated that bone turnover is increased in SHR due to *Opg/Rankl/Rank* elevation during PD since they indicate poor quality of the extracellular matrix, different from W animals. MCs depletion led to reduced expression of all axis components, suggesting that MCs play an important role in bone remodeling dynamics, as previously reported [53].

To further support this hypothesis, we analyzed bone resorption markers, including markers of osteoclast recruitment, activation, and activity. *Oscar* is an FcRγ-associated receptor that mediates co-stimulation of osteoclasts differentiation [54], and have also been associated with several bone loss conditions [55, 56]. An increase in *Oscar* expression in PD groups, with more prominent expression in SPD, was observed. However, previous MCs depletion did not modulate this response. The higher *Oscar* expression in SHR could be an intrinsic alteration and may not be attributed to the increased inflammatory status since *Oscar* expression remained unaltered even though MCs depletion reduced inflammatory mediator production. Further studies are required to better understand this difference between normotensive and hypertensive strains.

Integrins function as *Vtn* receptors and mediate cell adhesion, migration, and cell-cell interactions in a variety of cells [57]. Only SPD showed a significant increase in *Vtn* expression, while *Itga5* and *Itgb5* were increased in W and SPD. Notably, animals depleted of MCs had lower expression of all three markers. Lakkakorpi, Horton [58] showed that although *Vtn* is not present in the osteoclast adhesion zone membrane, expression in other membrane locations possibly influences the osteoclast migration and permanence in the resorption site. Integrins have an important role in bone physiology, El Azreq, Arseneault [59] demonstrated a functional relationship between Th17-type immune response and α2β1 integrin in osteoclast activity. Functional studies of integrin subunits in the alveolar bone remain scarce. Specifically, the aspects evaluated in this study are absent in the literature, possibly due to the difficulties involved in generating knockout models, as stated by Larjava, Koivisto [57]. However, our data support the participation in PD-induced alveolar bone resorption and suggest a significant involvement of MCs in the modulation of these responses.

Subsequently, in addition to cell recruitment, bone organic matrix degradation and remodeling are crucial steps in PD progression [60, 61]. *Mmp9* expression was increased in both PD groups, but *Mmp2* was only increased in SPD. MCs depletion was able to regulate the expression of these enzymes in a strain-specific manner between W and SHR, with a reduction in *Mmp2* and *Ctsk*, respectively, while Mmp-9 was not altered. SHR presents a lower matrix quality production, reflecting a more fragile mineral structure [30]. This effect, in combination with an increased expression of matrix enzymes, explains the greater susceptibility to bone destruction.

To study the effects on osteoclast maturation and activity, we analyzed *Trap* and *Ctsk* expression, two of the main bone resorption markers [61–63]. PD groups showed increased expression of both osteoclast markers, as previously reported [64, 65]. Increased *Trap* expression was significantly reduced in MCs depleted groups. Biosse-Duplan, Baroukh [66] reported the involvement of histamines, a major MCs-derived soluble mediator, in the recruitment and differentiation of TRAP-positive osteoclasts in rat mandibles, suggesting a possible mechanism that explains this response. PD induces an increase in *Ctsk* expression and is highly expressed in osteoclasts, contributing to the degradation of the extracellular matrix and alveolar bone resorption [67]. Interestingly in the current study, *Ctsk* expression was reduced in S48/80+PD, while it remained unaltered in W48/80+PD, compared to WPD. MCs depletion reversal was more significant in the SHR, which could be explained by the *Ctsk* response [68], but was not present in the W group, although the underlying mechanism of the *Ctsk* unaltered response remains unclear.

These results suggest that MCs modulate the PD-induced alveolar bone resorption, especially in the SHR, which is associated with a more severe PD progression compared to W. This can be partly explained by lower inflammatory mediator production that led to a reduced expression of osteoclast-related response markers. In addition, our data suggest that MCs response modulation could be a potential therapeutic approach to inflammation-driven bone resorption disorders.

## Supporting information

S1 Fig Absent of alveolar bone loss in Wistar and SHR (10-week-old) after 15 days from c48/80 treatment. Hemimandibles cleaned from soft tissue, further defleshed in 5% hydrogen peroxide solution for 24 hours and stained in 0.5% methylene blue solution for 30 seconds. The specimens were photographed in 1.5x magnification in a stereomicroscope (Olympus; SZ61), and the area from the cementoenamel junction (CEJ) to the alveolar bone crest (ABC) in the first molar was measured using ImageJ software (v1.47, National Institutes of Health) (n = 6).
(TIF)

S2 Fig Immunolabeling for (A) BMP2, (B) OCN, and (C) BSP in the furcation region of the lower first molar of Wistar and SHR with PD, depleted of mast cells (48/80+PD). The image board show representative images from each experimental group (n = 5).
(TIF)
