# Supplementary figures and images for: Mast cells contribute to alveolar bone loss in Spontaneously Hypertensive Rats with periodontal disease regulating cytokines production
Source: PLoS One. 2021 Mar 4;16(3):e0247372. doi: 10.1371/journal.pone.0247372 (PMC7932174; doi:10.1371/journal.pone.0247372)

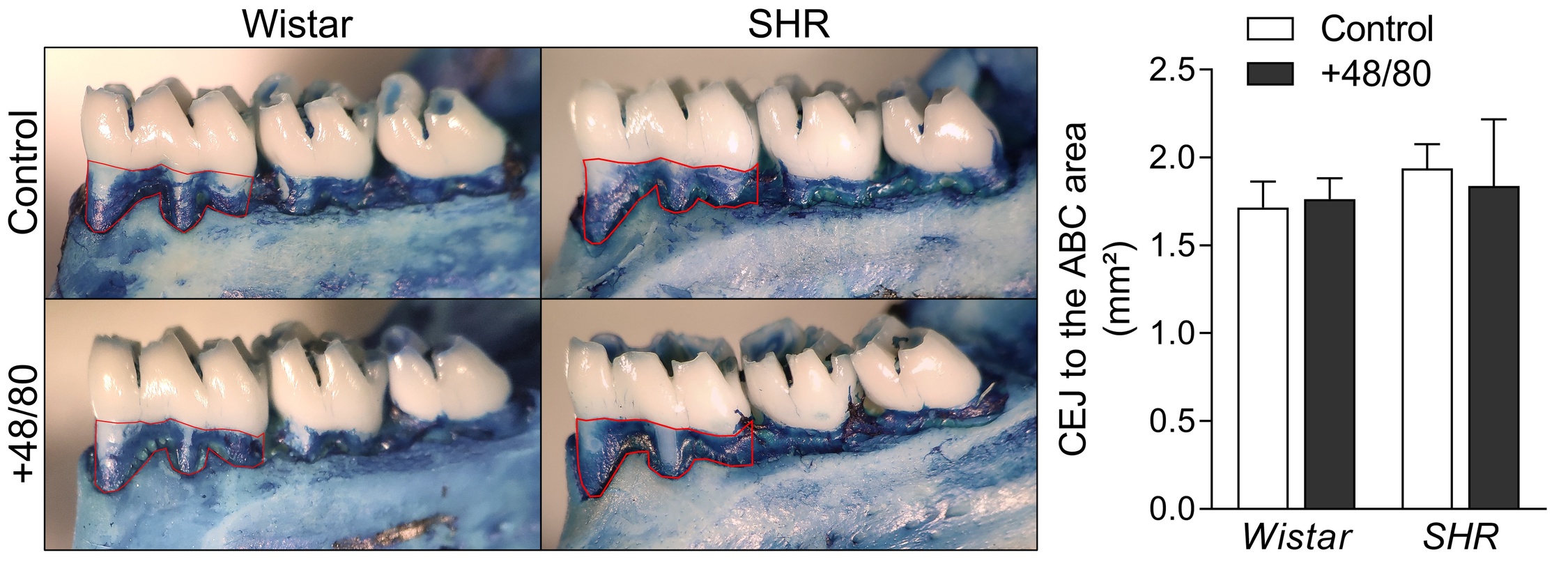

Supplement: S1 Fig — Hemimandibles cleaned from soft tissue, further defleshed in 5% hydrogen peroxide solution for 24 hours and stained in 0.5% methylene blue solution for 30 seconds. The specimens were photographed in 1.5x magnification in a stereomicroscope (Olympus; SZ61), and the area from the cementoenamel junction (CEJ) to the alveolar bone crest (ABC) in the first molar was measured using ImageJ software (v1.47, National Institutes of Health) (n = 6). (TIF) [file pone.0247372.s001.tif]

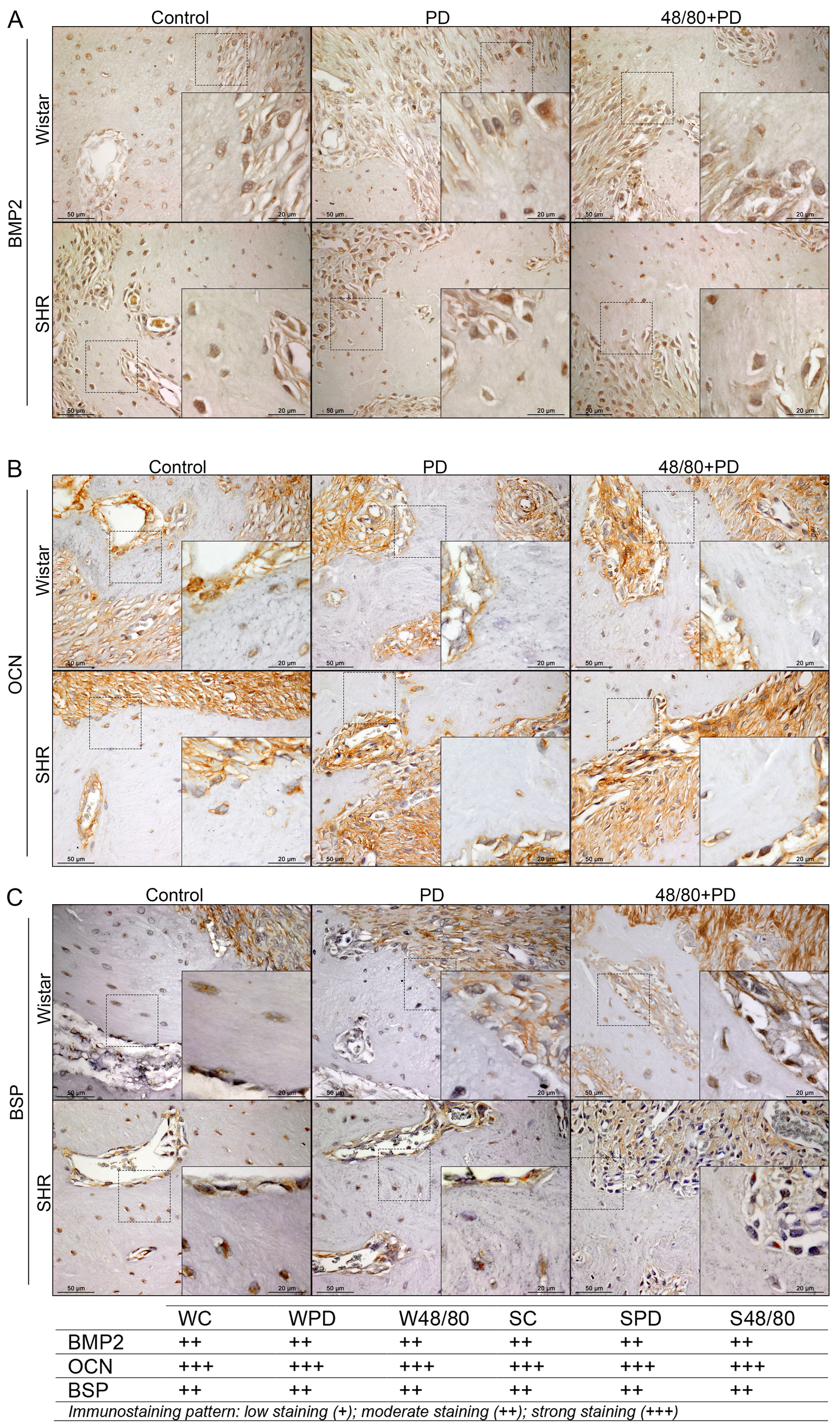

Supplement: S2 Fig — Immunolabeling for (A) BMP2, (B) OCN, and (C) BSP in the furcation region of the lower first molar of Wistar and SHR with PD, depleted of mast cells (48/80+PD). The image board show representative images from each experimental group (n = 5). (TIF) [file pone.0247372.s002.tif]
